# Fluorescence in situ hybridization (FISH): an increasingly demanded tool for biomarker research and personalized medicine

**DOI:** 10.1186/2050-7771-2-3

**Published:** 2014-02-05

**Authors:** Linping Hu, Kun Ru, Li Zhang, Yuting Huang, Xiaofan Zhu, Hanzhi Liu, Anders Zetterberg, Tao Cheng, Weimin Miao

**Affiliations:** 1 State Key Laboratory of Experimental Hematology, Institute of Hematology and Blood Diseases Hospital, Chinese Academy of Medical Sciences and Peking Union Medical College, Tianjin, China; 2 Department of Pathology, Institute of Hematology and Blood Diseases Hospital, Chinese Academy of Medical Sciences and Peking Union Medical College, Tianjin, China; 3 Department of Pediatrics, Institute of Hematology and Blood Diseases Hospital, Chinese Academy of Medical Sciences and Peking Union Medical College, Tianjin, China; 4 Tianjin Medical University Cancer Institute and Hospital, National Clinical Research Center of Cancer, Tianjin, China; 5 Department of Oncology-Pathology and Karolinska Cancer Center, Karolinska Institute, Stockholm, Sweden; 6 Center for Stem Cell Medicine, Chinese Academy of Medical Sciences and Peking Union Medical College, Nanjing Road 288, Tianjin 300020, P.R. China

**Keywords:** Fluorescence in situ hybridization (FISH), Solid tumors, Hematopoietic malignancies, Genetic aberrations, Biomarkers, Personalized medicine

## Abstract

Extensive studies of the genetic aberrations related to human diseases conducted over the last two decades have identified recurrent genomic abnormalities as potential driving factors underlying a variety of cancers. Over the time, a series of cutting-edge high-throughput genetic tests, such as microarrays and next-generation sequencing, have been developed and incorporated into routine clinical practice. Although it is a classical low-throughput cytogenetic test, fluorescence in situ hybridization (FISH) does not show signs of fading; on the contrary, it plays an increasingly important role in detecting specific biomarkers in solid and hematologic neoplasms and has therefore become an indispensable part of the rapidly developing field of personalized medicine. In this article, we have summarized the recent advances in FISH application for both de novo discovery and routine detection of chromosomal rearrangements, amplifications, and deletions that are associated with the pathogenesis of various hematopoietic and non-hematopoietic malignancies. In addition, we have reviewed the recent developments in FISH methodology as well.

## Introduction

Mounting evidence indicates that both hematologic and solid tumors are heterogeneous disorders with diverse genomic aberrations [1-3]. Due to the extensive investigations of the correlation between genomic instability and disease pathogenesis conducted over the last two decades, an increasing number of genomic abnormalities such as gain, loss or rearrangement of chromosomal fragments and gene mutations, have been found to be driving factors in the pathogenesis of various malignancies. Over the time, a series of cutting-edge cytogenetic and molecular tests have been developed for detecting such genomic aberrations, which allows more accurate molecular profiling for individual patients. The advanced molecular pathology techniques [4] enable better disease stratification and prognosis, leading to tailored therapeutic regimens. Apparently, a new era of personalized medicine has arrived much earlier than most of us expected [3].

Fluorescence in situ hybridization (FISH) is a cytogenetic technique developed in the early 1980s. FISH uses fluorescent DNA probes to target specific chromosomal locations within the nucleus, resulting in colored signals that can be detected using a fluorescent microscope. Compared to the conventional cytogenetic (CC) metaphase karyotype analysis, FISH does not require cell culturing, and can directly use fresh or paraffin-embedded interphase nuclei for a rapid evaluation. With the discovery of numerous disease-related genes in recent years, the applications of FISH broadened to include more genetic diseases, hematologic malignancies, and solid tumors. For example, FISH detection of BCR/ABL1 translocation, HER2 amplification, and ALK rearrangement is critical for guiding targeted therapy in chronic myeloid leukemia [5], breast cancer [6,7] and lung adenocarcinoma, respectively [8,9]. Hence, FISH tests have been recognized as vital components of personalized medicine.

With respect to biomarker detection, a series of innovative high-throughput molecular tests, such as array-based comparative genome hybridization (aCGH), single nucleotide polymorphism (SNP) arrays, and next generation sequencing, have recently been developed and incorporated into routine clinical practice. The stunning technology replacing speed raised a serious question: is a classical low-throughput assay, such as FISH, poised for replacement? However, the answer is quite the opposite. In fact, FISH has become increasingly important in clinical diagnosis due to its simplicity and reliability in evaluating key biomarkers in various tumors [5]. In this article, we aim to review the advances in FISH for disease biomarker detection and personalized medicine applications.

### Hematopoietic malignancies

#### Leukemia

Leukemia is a heterogeneous clonal disorder of hematopoietic stem and progenitor cells, characterized by various acquired genetic aberrations. The discovery of abnormal fusion proteins resulting from chromosomal rearrangements has significantly contributed to our understanding of the molecular mechanism of the pathogenesis of leukemia. The first oncogene discovered as the direct etiological basis of a malignancy, the BCR/ABL1 translocation in chronic myeloid leukemia (CML), results in dysregulated tyrosinase activity, which can be treated using the tyrosine kinase inhibitor Imatinib [10,11]. The t (15; 17) chromosomal translocation in promyelocytic leukemia (APML, AML-M3) functions in a similar way, generating the novel fusion protein PML/RARa, and ATRA (all-trans retinoic acid) offers an effective therapy for APML by specifically suppressing oncogenic activities of the PML/RARa fusion protein [12,13]. The FISH assay is considered the gold standard for detecting these chromosomal translocations and it therefore plays a crucial role in selecting a targeted therapy for various leukemias.

Over the last two decades, additional recurrent genetic aberrations have been identified in leukemias, improving molecular sub-classification and allowing stratified management. Whereas most of the chromosomal rearrangements can be detected using CC, FISH remains the most robust tool for detecting balanced or unbalanced chromosomal aberrations. Furthermore, a combination of cytogenetic and molecular profiling permits more accurate assessment of the disease prognosis [1].

In addition to the common chromosomal rearrangements in acute myeloid leukemia (AML), which are t(8;21)(q22;q22), t(15;17)(q22;q12) and inv(16)(p13.1q22) or t(16;16)(p13.1q22) [14], newly obtained molecular information has been combined with cytogenetic findings to establish a more comprehensive risk assessment system. Among them, t(8;21)(q22;q22)/RUNX1-RUNX1T1; t(15;17)(q22;q12)/PML-RARa; inv (16)(p13.1q22) or t(16;16)(p13.1;q22)/CBFB-MYH1 and mutation in both the CEBPA and NPM1 genes are associated with a good clinical outcome, whereas t(1;22) (p13;q13)/RBM15-MKL1; inv(3)(q21q26.2) or t(3;3)(q21;q26.2)RPN1-EVI1; t(6;9)(p23;q34)/DEK-NUP214, the MLL gene rearrangement, complex karyotypes, and mutations in both KIT and FLT3 are associated with a less favorable prognosis [14,15].

Acute lymphoblastic leukemia (ALL) occurs mostly in children between the ages of 1 to 5 years old. The most common chromosomal translocations in ALL are t (9; 22) (p190) in adults, and t (12; 21) in children respectively. The most frequent numerical aberration in ALL is hyperdiploidy with chromosome numbers ranging from 51 to 63, with chromosomes X, 4, 6, 10, 14, 17 and 18 generally being trisomic and chromosome 21 frequently occurring as four copies [16,17]. Among them, patients with t (12; 21), t (1; 19), and hyperdiploidy have a favorable outcome, whereas patients with MLL translocations have a worse prognosis. It is worth noting that the presence of t (9; 22) used to indicate the worst prognosis for ALL patients, but this fact has dramatically changed since the introduction of the “magic bullet” Gleevec.

Due to the universal presence of BCR/ABL1 rearrangement in chronic myeloid leukemia (CML), it is now defined as the diagnostic hallmark of CML [10,11,18].

Chronic lymphocytic leukemia (CLL) generally presents as an indolent disorder but can be aggressive in some patients due to various genetic aberrations. The most common recurrent chromosomal abnormalities are trisomy 12, del(13q), del(11q), del(17p) and del(6q) [19-21]. FISH can identify chromosomal rearrangements in approximately 80% of patients, whereas CC can identify chromosomal aberrations in only approximately 40-50% patients. The genetic information provided by FISH tests can be critical for therapeutic decisions. Chemoimmunotherapy using fludarabine, cyclophosphamide and rituximab (FCR) receives a better treatment response from patients with trisomy 12 or del(11q), whereas patients with 17p deletions do not benefit from FCR treatment at all [20,22]. In addition to the therapeutic significance, FISH tests facilitate differential diagnosis between CLL and other types of small B-cell lymphoma/leukemia. For example, mantle cell lymphoma is morphologically similar to CLL, but carries a characteristic genetic aberrancy, the cyclin D1 translocation, and has a much worse prognosis. The FISH test is the gold standard method of identifying the cyclin D1 rearrangement, particularly in when immunohistochemistry is not contributory for various reasons.

#### Multiple myeloma

Multiple myeloma (MM) is another heterogeneous malignancy of terminally differentiated B cells, clinically manifested as monoclonal plasma cells that infiltrate the bone marrow, a spike of monoclonal immunoglobulin in the blood and/or urine, and massive osteolytic bone lesions. Similar to the other hematologic neoplasms, MM is characterized by a complex pattern of extensive genomic aberrations involving many chromosomes [23,24]. The genetic abnormalities found in MM can be roughly divided into two categories based on the chromosome ploidy status and other parameters [25,26]. The hyperdiploid karyotype is generally associated with trisomies of many chromosomes, such as 3, 5, 7, 11, 15, 19 and 21, whereas the hypodiploid karyotype appears to be more frequently associated with a translocation of immunoglobulin heavy chain (IGH) locus at 14q32 [26]. The IgH (14q32) translocations found in hypodiploid MM can involve many different partners, such as 11q13 (CCND1), 6p21 (CCND3), 16q23 (MAF), 20q12 (MAFB), and 4p16 (FGFR3 and MMSET). Furthermore, the chromosome ploidy status and IGH rearrangements were found to be correlated with disease outcome in MM patients [25]. For example, the hyperdiploid karyotype with t(11;14)(q13;q32) indicates a better prognosis, whereas the hypodiploid karyotypes with t(4;14)(p16; q32) or t(14;16)(q32;q23) imply a worse clinical outcome [25,26].

Molecular studies have demonstrated that primary translocations occur in the early stage of MM, followed by large number of secondary translocations during tumor progression [27]. It is believed that the secondary genomic aberrations are responsible for a more proliferative phenotype in the advanced stage of MM. Certain genetic aberrations, such as MYC rearrangements, del (13q), del (17p), and the deletion of 1p and/or amplification of 1q, have been identified as the most common secondary aberrations in MM [27-29]. The chromosome 13 deletion or chromosome 13 monosomy occurs in 50% of the patients with advanced MM and are associated with an aggressive clinical course and an unfavorable prognosis [30,31]. Deletion of 17P13, presumably resulting in LOH (loss of heterozygosity) of P53, has been determined to be associated with a very poor clinical outcome [32,33]. Chromosome 1p deletion or 1q amplification is the most common structural aberration found in MM and is associated with an unfavorable prognosis [34-36].

Due to the low proliferative rate of tumor cells in the early stage of MM, CC analysis of metaphase cells is likely to miss detecting the primary genomic aberrations in non-dividing tumor cells. Furthermore, some small chromosomal rearrangements in MM may be cryptic to chromosome banding analysis. Thus FISH, which is effective for analysis of interphase nuclei and small chromosomal aberrations, is recognized as the most robust genetic test for characterizing the known cytogenetic abnormalities in MM. Nevertheless, integration of data from the multiple genetic profiling techniques including CC, FISH, RT-PCR, and gene mutation analysis, among others, would provide comprehensive information for better stratification of MM patients with diagnostic and prognostic significance.

#### Myelodysplastic syndromes (MDS)

MDS is a heterogeneous group of clonal hematopoietic disorders characterized by blood cytopenias resulting from ineffective hematopoiesis. The clinical outcome of MDS is variable, and approximately 20 to 30% of the patients will progress to AML within a few months or years [37]. Recurrent chromosomal abnormalities, such as -5/del(5)(q31), -7/del(7)(q31), +8, del(20)(q2), -17/del(17)(p3.1), and –Y, are found in half of the de novo MDS cases. The cytogenetic finding is closely related to the clinical outcome, and thus has been incorporated into the revised international prognostic scoring system (IPSS-R) and the WHO prognostic scoring system (WPSS) [38]. In addition, certain chromosomal aberrations, such as -5/5q-, -7/7q-, and complex abnormalities, were found to be correlated to the response to chemotherapy [37,39,40]. The metaphase chromosomal banding assay is regarded as the objective standard for clonal analysis of MDS. Being a more sensitive test, FISH can be utilized to identify minor abnormal clones and cryptic chromosomal aberrations that are undetectable using CC and to provide additional information for patients with a normal karyotype or unsuccessful culture [5,39]. With the introduction of high-resolution assays such as aCGH and SNP arrays, it will be possible to discover additional genetic markers in the near future, leading to more accurate diagnoses, better targeted therapies, and more clinically significant judgment of the prognosis for MDS patients [41].

### Non-hematopoietic malignancies (solid tumors)

#### Lung cancer

Anaplastic lymphoma kinase (ALK) rearrangements, which are generally associated with pulmonary adenocarcinomas in female non-smokers, occur in approximately 5% of patients with non-small cell lung cancer (NSCLC). ALK rearrangements mostly result from the fusion of the echinoderm microtubule-associated protein-like 4 (EML4) with ALK at chromosome 2p23. Fusion of ALK kinase with EML4 or other fusion partners, such as TFG or KIF5B, leads to constitutive activation of ALK kinase [39,42]. Patients with EML4-ALK fusion-positive NSCLC, who were treated with the small-molecule kinase inhibitor Crizotinib, showed a response rate of 50-60% [8]. 2011 was the first time that the US FDA simultaneously approved a novel anti-cancer drug (Crizotinib, Pfizer) and its companion FISH detection kit (ALK FISH probe kit, Abbott Molecular), which highlighted the critical role of the FISH assay in guiding ALK-targeted therapy [8,9]. Because ALK rearrangements are reportedly mutually exclusive with EGFR/KRAS mutations [43], ALK FISH testing is generally recommended for patients with wild-type EGFR/KRAS non-squamous NSCLC.

Using the ALK FISH detection kit, the 3’ and 5’ ends of the ALK gene are differentially labeled with red or green fluorescent probes. In benign cells, two fused signals should be detected. In 60-70% of all of the ALK rearrangements, the EML4-ALK gene fusion occurs through only inversion, and therefore narrowly separated (two to three signals apart) red and green signals are detected in addition to the normal fusion signal. In the other 30-40% of the cases, gene fusion occurs through an interstitial deletion together with an inversion of EML4, which lead to a single red signal without a corresponding green signal, in addition to the normal fusion signal [44].

ROS1, another receptor tyrosine kinase, which is located at chromosome 6p22, has recently been found to be rearranged in 2-3% of the NSCLC adenocarcinomas [7,45]. In addition, several ROS1 translocation partners, e.g., TPM3, SDC4, SLC34A2, CD74, EZR or LRIG3, have been found forming a fusion with the kinase domain of ROS1, leading to constitutive activation of ROS1 and increase in malignant transformation activity [7]. The ROS1 rearrangements define a subset of NSCLC with clinical characteristics and treatment responses that are similar to those of the ALK rearrangements [45,46]. Therefore, this type of rearrangement is another predictive FISH biomarker that can be applied to personalized management of lung cancer.

#### Prostate cancer

Rearrangements involving androgen-regulated TMPRSS2 and ETS family members (ERG, RTV1, ETV4) were detected in nearly half of the prostate cancers but none of the benign prostate tissues that were tested [5,39]. The relevance of the TMPRSS2 rearrangements to the pathogenesis, prognosis, and targeted therapy of prostate cancer has made it a predictive biomarker for prostate cancer [47]. The most common type of chromosome rearrangement involves the fusion of TMPRSS2 to the oncogene ERG, which leads to the abnormal activation of ERG. Identification of these rearrangements may allow stratification of prostate cancers into subtypes that respond to specific therapies. As the test of choice for chromosomal rearrangements, FISH was successfully applied to frozen as well as formalin-fixed paraffin-embedded (FFPE) prostate cancer samples with high sensitivity and specificity. Initially, a kit containing dual-color ERG break-apart probes that can identify rearrangement of the ERG gene but do not indicate the 5’-partner to which ERG is fused to, was used [48,49]. Later on, a tricolor FISH assay was developed by combining the red/green break-apart probes for TMPRSS2 with an orange-labeled fusion probe for the 3’ region of ERG [50,51]. Recently, a four-color FISH assay was reported, which allows the detection of either TMPRSS2 or ERG rearrangements regardless of the partner gene [39].

#### Breast cancer

Breast cancer is a fairly heterogeneous malignancy that involves large numbers of genomic aberrations that are inherited or are acquired during the initiation and progression of the disease. To date, the most successful application of FISH as a companion diagnostic test for selecting a targeted therapy for a solid tumor may be the FISH evaluation of Her-2 amplification for breast cancer [52-54]. Her-2 (human epidermal growth factor receptor-2), also called c-erbB-2, is located at chromosome 17q12-21.32, and encodes a trans-membrane protein of 185 kDa. Her-2 protein is an active tyrosine kinase that plays an important role in normal cell growth and differentiation. It has been reported that Her-2 gene amplification occurs in 20-30% of breast cancer patients. Her-2 gene amplification leads to its overexpression on the cell surface. Her-2 amplification indicates a bad prognosis, short survival time, and the existence of a more aggressive phenotype of tumor cells. Her-2 overexpressed breast cancer may be resistant to endocrine therapy and some chemotherapies; however, it is sensitive to Herceptin treatment and exhibits more responsiveness to paclitaxel and anthracyclines [55]. At present, there are both IHC and FISH assays for measuring Her-2 overexpression. The former assay is simplistic, convenient, and cost-saving, but it can be affected by various factors and the staining result could be ambiguous. The latter is relatively sophisticated and expensive, but the staining result is more accurate. Therefore, the FISH assay is regarded as the gold standard for clinical evaluation of the Her-2 status and is generally recommended in when the IHC result could not clarify the Her-2 status.

Traditionally, breast cancer is classified as high risk and low risk based on the tumor’s size, grade, nodal and ER status. However, it is noted that 15% of patients with low risk parameters (tumor size <1 cm, low grade, lymph node negative, ER positive) have recurrent disease and usually die of metastasis. Meanwhile, 15% of patients in the high risk group (tumor size > 5 cm, high grade, lymph node positive, ER negative) have unexpected favorable clinical outcome. These patients could receive mistreatments based on their histopathological classifications; therefore, there is a need to establish more accurate molecular classification schemes [56]. A molecular classification based on a clustering analysis of the expression patterns of 427 genes has divided breast cancer into four types: a luminal type (further divided into A, B and C subtypes), a basal-like type, a Her-2 positive type, and a normal breast-like type [57,58], and the molecular classifications are closely correlated with the prognoses, with the luminal subtype A having a good clinical outcome; the luminal subtype B having a bad prognosis; and the basal-like and the Her-2 positive types having the worst clinical outcome [58]. Recent CGH analyses of genomic aberrations in breast cancer have identified three molecular categories of breast cancers. The first category, called “simplex” [59] or 1q/16q [60,61], is characterized by a few genomic rearrangements and the second category, called “complex sawtooth” [59] or “complex” [60,61], is characterized by more rearrangements and gene copy number alterations within a restricted genomic area. The third category, called “complex firestorm” [59] or “mixed amplifier” [60,61], is characterized by high intensity gene amplification profiles restricted to a small genomic areas. Interestingly, correlations seem to exist between the previous Sorlie expression classes [58] and the specific genomic profile categories, possibly due to the interplay of the genomic aberrations and the overall gene expression. The luminal A type of breast cancer is correlated with the simplex profile and the luminal B and the Her-2 positive types with the complex firestorm profile. The basal-like class is correlated with the complex sawtooth profile [62].

The estrogen receptor (ER) is a well-established biomarker for endocrine therapy in breast cancer patients, while the progesterone receptor (PR) is not. Clinical studies showed that ER + PR + breast cancer shows a better response to endocrine therapy, whereas ER + PR- breast carcinoma has a more aggressive phenotype and a poorer response to endocrine therapy. CGH and FISH studies have revealed that ER + PR- breast cancers have higher genomic instability profiles, including recurrent amplifications in 11q13, 12q14-q15, 17q21-q25, and 20q13 and deletions in 11q13-q15 [60,63-65]. A more recent study refined the area to 17q23.2-q23.3 and 20q13.12 for most of the overlapping gained regions, and 3p21.32-p12.3, 9pter-p13.2, 17pter-p12, and 21pter-q21.1 for most of the overlapping lost areas in ER + PR- breast cancers [65].

Variations in the genomic profiles exist not only among different histopathological types of breast cancer, but are also found among the types of breast cancers of different ethnic groups. A recent study comparing breast cancer samples from African and American women has identified 6 chromosomal regions with a higher rate of CNAs and several candidate biomarkers that could be specific to African women [66].

Extensive screening of genomic aberrations has led to the identification of candidate biomarkers associated with breast cancer tumorigenesis, invasiveness, and metastasis, including MYC at 8q24 [67], CCND1 at 11q13 [68], Her-2 at 17q12 [53], MTDH at 8q22 [69,70], and ETS transcription factors at 1q21 and 1q32 [71].

While substantial progress has been made in breast cancer genetics over the last two decades, further large-scale studies integrating both genome and transcriptome analyses [72] are needed to identify the key oncogenic driver genes or other specific biomarkers. The potential new findings could be of predictive values for diagnosis, predicting metastasis, survival assessment, and guiding targeted therapy. FISH can be of particular value in both the discovery and clinical routine detection of such biomarkers and will continue to play an important role in the personalized management of breast cancer.

#### Melanoma

Melanoma is a heterogeneous group of melanin-producing skin malignancies with acquired genetic aberrations. Studies employing aCGH and FISH have identified a variety of recurrent chromosomal aberrations in malignant melanoma. In clinic, a small but significant part of melanocytic lesions presents with ambiguous morphologic features, and those cases are challenging to experienced dermatopathologists. Thus, a specific ancillary genetic test is needed for the initial characterization to avoid misdiagnosis and overtreatment [73,74]. Most primary melanomas exhibit either numerical or structural chromosomal abnormalities, such as deletions in 9p, 10, 6q and 8p and copy-number increase in 7, 8, 6p, 1q, 20, 17, and 2 [75,76]. Given that multiple chromosomal aberrations must be evaluated to obtain genetic profiles of melanoma, a multi-color approach comprising 4 gene probes have been adopted for a FISH-based melanoma assay [77]. The clinical studies showed that use of FISH in unambiguous cases provided promising results with relatively high sensitivity and specificity. However, the diagnostic utility of FISH in ambiguous cases remains to be determined because a standard definition of “malignancy” is yet to be established from clinical studies with large samples of ambiguous cases [78]. Furthermore, the discovery of key genomic aberrations has led to more effective targeted therapies for melanoma. For example, Vemurafenib (BRAF V600E inhibitor) and Ipilimumab (anti-CTLA4) were recently approved by the U.S. FDA and agents directed against the MAP kinase pathway (anti-MEK, anti-ERK, other anti-BRAF) are under development for targeted therapy in cases of advanced metastatic melanoma [79].

### Progress in FISH methodology

#### FISH automation

Manual evaluation of large numbers of clinical FISH samples is no doubt a time-consuming, exhausting, and error-prone procedure. Moreover, the inter-observer variability could lead to scoring inconsistency and even misdiagnosis. Thus, an automated system would greatly reduce such errors. Such an automated system is generally comprised of the following parts: a light source, filter set, objectives, signal detector, motorized scanning stage, and a computer equipped with dedicated software in charge of automated tissue-area selection, signal evaluation and data calculation [80]. There were several reports of the successful automated evaluation of HER2 gene amplification using breast cancer specimens [81-83]. An automated method has also been applied to detecting balanced rearrangements, such as the BCR/ABL1 gene rearrangements in patients with CML [84]. Nevertheless, automated FISH is still in a premature stage with more standardization and large scale clinical trials pending.

#### QM-FISH

Previously, most commercial FISH detection kits contained one probe labeled with a single fluorochrome or two probes labeled with two distinct fluorochromes. These single or dual-color kits were used to detect a deletion or an amplification of a single locus-specific genomic fragment or a balanced chromosomal translocation. With the rapid progress in disease gene discoveries, there is a need to simultaneously detect multiple genes. Thus, a FISH method that employs multiple probes, called quantitative multi-gene FISH (qmFISH) has recently become popular. Abbott’s MultiVysion PB multi-color probe kit, a five-color FISH kit that detects chromosomes 13, 16, 18, 21 and 22 was developed to assist in preimplantation diagnosis (PGD) by polar body analysis [85]. A four-color FISH assay, targeting chromosomes 1, 2, 6, 9, 7, 17, the loci 3p24pter, and 3p13p14 has been used for the early diagnosis of renal carcinoma in biopsies of uncertain renal masses [86]. LAVysion FISH, a four-color FISH kit for simultaneously detecting chromosome 6 and the 5p15, 7p12 (EGFR gene), and 8q24 (MYC gene) loci was developed to assist in the differential diagnosis of ambiguous lung cancers [87]. In recent years, qmFISH has been used in genetic variegation and clonal evolution studies of both hematological and non-hematological cancers [88-92].

We have developed a state-of-the-art qmFISH system that can use as many as 10-20 fluorochromes, and the signals could only be analyzed by a computer-controlled detector (Zetterberg, A. et al, unpublished). This system is particularly useful for large-scale multi-gene clinical investigations of solid tumors or blood malignancies.

**Figure 1:**
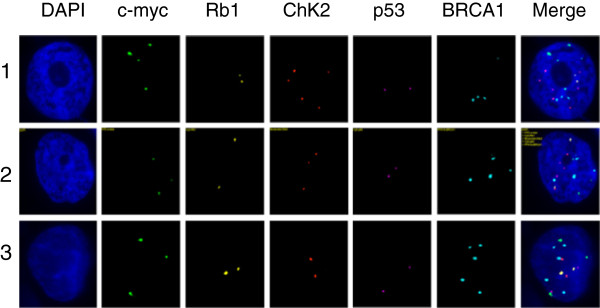
**The three subclones from the ascites cytospin sample of a progressive epithelial ovarian cancer patient.** BAC probes containing the c-myc, Rb1, Chk2, p53 or BRCA1 genes were labeled with Spectrum Green, PF555 (red), PF590 (orange), HyPer5 (purple) or PF415 (blue), respectively. The mixed probes were hybridized with the ascites cytospin sample from a progressive epithelial ovarian cancer patient. The results revealed that there were three subclones showing distinct combinations of signal patterns for the five selected genes. The details of the molecular profiling are shown in Table 1.

**Figure 2:**
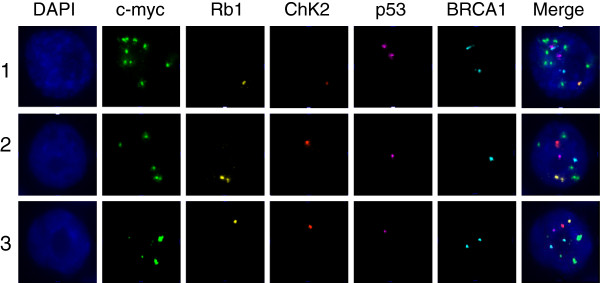
**The three subclones obtained from a cancer stem-cell preparation for a patient with low-differentiated ovarian adenocarcinoma.** BAC probes containing c-myc, Rb1, Chk2, p53 or BRCA1 were labeled with Spectrum Green, PF555 (red), PF590 (orange), HyPer5 (purple) or PF415 (blue), respectively. The mixed probes were hybridized with the cancer stem-cell preparation sample from a low-differentiated ovarian adenocarcinoma patient. The results revealed that there were three subclones showing distinct combinations of signal patterns for the five selected genes. The details of the molecular profiling are shown in Table 2.

Currently, we are applying qmFISH to study the genetic architecture and clonal evolution in cases of ovarian cancer and leukemia. Ovarian cancer is a heterogeneous female malignancy characterized by various genomic aberrations. To define the molecular subgroups of ovarian cancer, we have chosen five genes, c-myc, Rb1, Chk2, p53 and BRCA1, which are known to be associated with the pathogenesis of ovarian cancer. The ascites fluid samples were collected from ovarian cancer patients from Tianjin Medical University Cancer Institute and Hospital from January to December 2012, with hospital ethical review committee approval. The ovarian cancer cell sections were prepared by Cytospin procedure and fixed overnight in methanol. qmFISH was performed as previously described [93]. In each case, at least 200 nuclei were scored for CNAs (copy number alterations) of c-myc, Rb1, Chk2, p53 and BRCA1. The results showed that an ascites fluid cytospin sample from a representative case of progressive epithelial ovarian cancer contained at least three subclones with distinct molecular profiles for the selected genes (Figure 1 and Table 1). Furthermore, a CD133 + ALDH + cancer stem cell [94,95] preparation isolated from a case of low-differentiated ovarian adenocarcinoma included three subclones with distinct qmFISH fingerprints (Figure 2 and Table 2). Thus, qmFISH analysis appears to be an excellent tool for molecular profiling of ovarian cancer at the single cell level.

**Table 1:** The three subclones in an ascites fluid sample from a patient with progressive epithelial ovarian cancer

**Clone**	**c-myc**	**Rb1**	**Chk2**	**P53**	**BRCA1**
1	4	2	5	2	4
2	3	2	3	2	4
3	3	2	2	2	5

Note: the numbers in Clone column indicate the coding number of the subclones; the numbers in FISH probe columns (c-myc, Rb1, Chk2, p53 and BRCA1) indicate copy numbers of each gene within a single nucleus of the cancer clone.

**Table 2:** The three subclones obtained from a cancer stem-cell preparation for a patient with low-differentiated ovarian adenocarcinoma

**Clone**	**c-myc**	**Rb1**	**Chk2**	**P53**	**BRCA1**
1	9	1	1	2	2
2	5	2	1	1	1
3	4	1	1	1	2

Note: the numbers in Clone column indicate the coding number of the subclones; the numbers in FISH probe columns (c-myc, Rb1, Chk2, p53 and BRCA1) indicate copy numbers of each gene within a single nucleus of the cancer clone.

**Figure 3:**
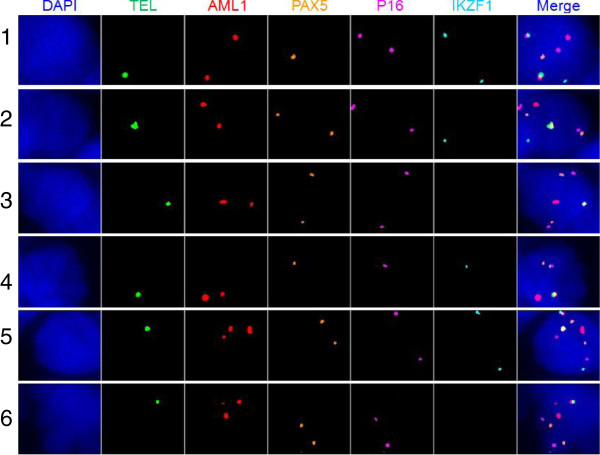
**The six subclones from the bone marrow sample taken from an ALL patient.** BAC probes containing the TEL, AML1, PAX5, p16 or IKZF1 genes were labeled with SpectrumGreen, PF555 (red), PF590 (orange), HyPer5 (purple) or PF415 (blue), respectively. The mixed probes were hybridized with the bone marrow sample of an ALL patient. The results revealed that there were six subclones showing distinct combinations of the signal patterns for the five selected genes. The details of the molecular profiling are shown in Table 3.

**Figure 4:**
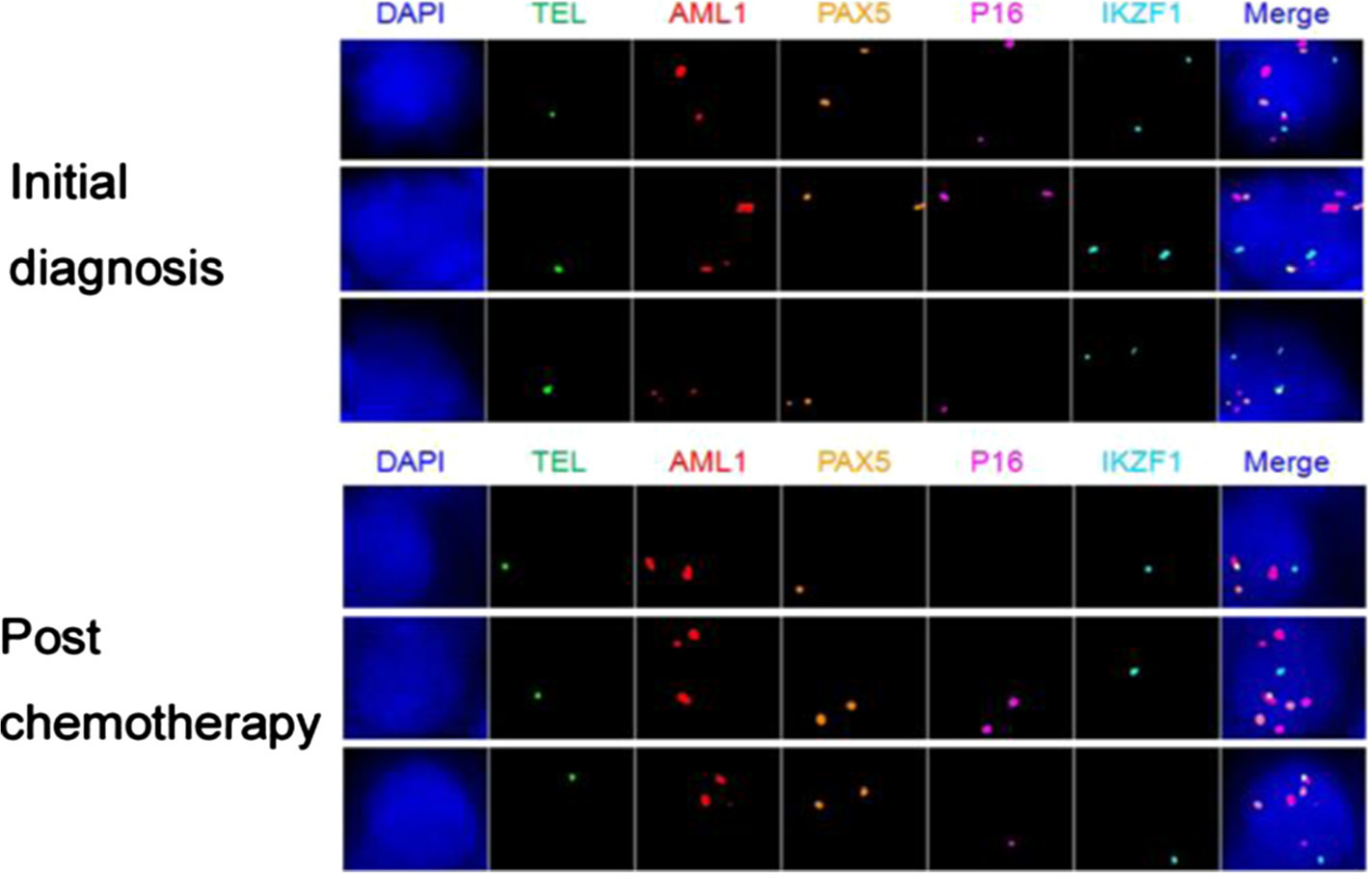
**The clonal components of bone marrow samples taken from an ALL patient upon the initial diagnosis and after chemotherapy.** BAC probes containing the TEL, AML1, PAX5, p16 or IKZF1 genes were labeled with Spectrum Green, PF555 (red), PF590 (orange), HyPer5 (purple) or PF415 (blue), respectively. The mixed probes are hybridized with the bone marrow samples upon the initial diagnosis and after thermotherapy of an ALL patient. The results revealed that there are distinct subclones upon the initial diagnosis and after chemotherapy, which showed different combinations of signal patterns for the five selected genes. The details of the molecular profiling are shown in Table 4.

Another of our projects is to apply qmFISH to molecular profiling of acute lymphatic leukemia (ALL). It has been reported that t (12; 21) leads to fusion of an almost complete RUNX1(AML1) protein to part of the ETV6 (TEL) protein, which is found in 20-25% of the ALL patients. The ALL patients with the ETV6 (TEL)/RUNX1(AML1) translocation generally have a better clinical outcome, but are more likely to experience a recurrence. The deletion of ETV6 (TEL) gene, which is associated with the ETV6 (TEL)/RUNX1(AML1) translocation, is common in ALL and leads to LOH of 12p12-13. In addition to ETV6 (TEL) and RUNX1(AML1), we selected PAX5, CDKN2A(P16) and IKZF, which are known to be associated with ALL pathogenesis, as target genes for the molecular profiling [88,96]. The bone marrow (BM) samples were collected from the ETV6 (TEL)/RUNX1(AML1) positive ALL patients from Tianjin Blood Diseases Hospital from January to December 2012, with hospital ethical review committee approval. The BM mononuclear cells were fixed in methanol:acetic acid solution. qmFISH was performed as previously described [93]. In each case, at least 200 nuclei were scored for the presence of the ETV6 (TEL)/RUNX1(AML1) fusion gene in combination with CNAs of ETV6 (TEL), RUNX1(AML1), PAX5, CDKN2A(p16) and IKZF. The results showed that the bone marrow sample of a representative case of ALL contained at least 6 subclones with distinct combinations of molecular patterns of the five selected genes (Figure 3 and Table 3). The qmFISH studies have also been successfully used to compare the clonal components of bone marrow samples taken from the same ALL patient upon the initial diagnosis and post chemotherapy, which demonstrated a clonal evolution phenomenon (Figure 4 and Table 4). Taken together, our results showed that qmFISH is a useful tool for analyzing the genetic architecture and clonal evolution of leukemia cells, which could provide important information for monitoring the disease process and appropriately selecting the therapy.

**Table 3:** The six subclones from the bone marrow sample taken from an ALL patient

**Clone**	**Fusion**	**TEL**	**AML1**	**PAX5**	**P16**	**IKZF1**
1	1	1	2	1	2	2
2	1	1	2	2	2	1
3	1	1	2	2	2	0
4	1	1	2	1	1	1
5	1	1	3	2	2	2
6	1	1	3	2	2	0

Note: the numbers in Clone column indicate the coding number of the subclones; the numbers in FISH probe columns (Fusion, TEL, AML1, PAX5, p16 and IKZF1) indicate copy numbers of each gene (or gene fusion) within a single nucleus of the cancer clone.

**Table 4:** The clonal components of bone marrow samples taken from an ALL patient upon the initial diagnosis and post chemotherapy

**Clone**	**Fusion**	**TEL**	**AML1**	**PAX5**	**P16**	**IKZF1**
Initial diagnosis	1	1	2	2	2	2
	1	1	3	2	2	2
	1	1	3	2	1	2
Post chemotherapy	1	1	2	1	0	1
	1	1	3	2	2	1
	1	1	3	2	1	1

Note: the numbers in FISH probe columns (Fusion, TEL, AML1, PAX5, p16 and IKZF1) indicate copy numbers of each gene (or gene fusion) within a single nucleus of the cancer clone.

## Concluding remarks

While high-resolution molecular profiling techniques such as aCGH, SNP array analysis and whole-genome sequencing play critical roles in identifying novel chromosomal abnormalities, they are not practical for a routine application of clinical diagnosis due to various reasons. In contrast, FISH continues to work as a cornerstone in genetic labs due to its specificity, simplicity and reliability. Given the theory that cancer may be derived from tumor stem cells [97], the genetic abnormalities detected by FISH are likely to represent the initial or crucial genetic lesions responsible for cancer at the stem-cell level. Currently, FISH is widely used for detecting specific genomic aberrations, providing important information for disease diagnosis, risk stratification and prognosis (Tables 5 and 6). Furthermore, FISH is the gold standard for evaluating some key biomarkers, such as BCR/ABL1, HER2 and ALK rearrangements, and plays a critical role in guiding targeted therapies. In conclusion, FISH has evolved to become a vital diagnostic tool for personalized medicine.

**Table 5:** FISH probes that are commonly used for clinical diagnosis of hematological diseases

**Probes**	**Cytogenetic anomaly**	**Associated disorders**
BCR/ABL	t(9;22)(q34;q11)	CML, ALL, AML-M1, AML-M2, MPD
PML/RARα	t(15;17)(q22;q21)	AML-M3, CML Ph+
AML1/ETO	t(8;21)(q22;q22)	AML-M2, AML-M4, MDS
MLL (11q23)	t(1;11)(p32;q23)	ALL, AML
	t(1;11)(q21;q23)	AML-M4, AML-M5
	t(2;11)(p21;q23)	MDS
	t(4;11)(q21;q23)	ALL, AML
	t(6;11)(q27;q23)	AML-M4, AMl-M5
	t(9;11)(p22;q23)	ALL, AML-M5, MDS, t-MDS
	t(10;11)(p13;q23)	AML-M4, AMl-M5
	t(11;17)(q23;q21)	AML-M3, AML-M4, AMl-M5
	t(11;19)(q23;p13)	ALL, AML-M4, AML-M5, t-AMl
	t(X;11)(q13;q23)	T-ALL
	del(11q23)	AML, ALL, CLD, CLL, MDS, NHL
CBFB (16q22)	t(16;16)(p13;q22)	AML-M4Eo, MDS
	inv(16)(p13q22)	AML-M4Eo
	del(16q22)	AML, AML-M4Eo, NHL
EVI1 (3q26)	t(3;3)(q21;q26)	AML, MDS
	inv(3)(q21q26)	AML-M4, AML-M6, CML Ph+, MDS
	t(3;21)(q26;q22)	AML, CML Ph+, MDS
FGFR1/D8Z2 (8p11)	t(8;13)(p11;q12)	MPD
	t(8;16)(p11;p13)	AML-M4, AML-M5
TEL/AML1	t(12;21)(p13;q22)	ALL
TCF3/PBX1	t(1;19)(q23;p13)	pre-B ALL
CKS1B (1q21)/CDKN2C (1p32)	dup(1)(q21q32)	ALL, CLD, NHL
	del(1)(q21)	NHL
	del(1)(p32p36)	CLD, NHL
MYC (8q24)	t(2;8)(p12;q24)	ALL-L3, BL, NHL
	t(8;14)(q24;q32)	ALL-L3, BL, MM, NHL
	t(8;14)(q24;q11)	T-ALL
	t(8;22)(q24;q11)	ALL-L3, BL
CEP8	+8	ALL, AML, CLD, MPD, MDS, PV
EGR1 (5q31)/D5S721 (5p15.2)	-5	AML, MDS
	del(5)(q13q33)	AML, MDS, MPD, 5q- syn
D7S486 (7q31)/CEP7 (7p11.1-7q11.1)	-7	AML, MDS, MPD
	del(7)(q11)	ALL, AML, MDS
	del(7)(q22q34)	AML, CLD, CMD, MDS, NHL
D20S108 (20q12)	del(20)(q11q13)	AML, CMD, MDS, PV
	-20	ALL
RB-1 (13q14)	del(13)(q12-q22)	AML, AMM, CLD, CLL, MM, MDS, NHL
	del(13)(q12-q14)	AML, AMM, CLD, MDS, NHL
P53 (17p13.1)	del(17)(p13.1)	ALL, AML, CLD, MDS, NHL
	-17	CLL
IGH (14q32)	del(14q32)	CLD, NHL
	t(2;14)(p13;q32)	B-CLL
	t(3;14)(q27;q32)	DLCL, FL
	t(4;14)(p13;q32)	MM
	t(5;14)(q31;q32)	ALL
	t(6;14)(p25;q32)	MM
	t(8;14)(q24;q32)	ALL
	t(9;14)(p12-13;q32)	B-NHL, LPL
	t(11;14)(q13;q32)	B-PLL, CLD, MM, MCL, MGUS, NHL
	t(14;16)(q32;q23)	MM
	t(14;18)(q32;q21)	CLD, FL, DLCL, MM, NHL
	t(14;19)(q32;q13)	CLD, CLL, NHL
	t(14;22)(q32;q11)	ALL
ATM (11q22.3)	del(11)(q13q14-q23)	AML, CLD, CLL, MDS, NHL
FGFR3/IGH	t(4;14)(p13;q32)	MM
MAF/IGH	t(14;16)(q32;q23)	MM
CCND1/IGH	t(11;14)(q13;q32)	B-PLL, CLD, MM, MCL, MGUS, NHL
CEP12	+12	AML, CLL, CLD, NHL
BCL6 (3q27)	t(3;14)(q27;q32)	DLCL, FL
	t(3;22)(q27;q11)	DLCL, FL
BCL2 (18q21)	t(11;18)(q21;q21)	MZL, NHL
	t(14;18)(q32;q21)	CLD, FL, DLCL, MM, NHL
	del(18)(q21)	AML, CLD, NHL
	-18	CLD
	+18	ALL, CLD
CEPX/Y	XY/XX	ALLO-SCT
	-Y	ALL, AML, CLD, MDS, MM, MPD, NHL, PV

**Table 6:** FISH probes that are commonly used for clinical diagnosis of solid tumors

**Probes**	**Cytogenetic anomaly**	**Associated disorder**
CEP3,7,17; p16	Chromosome 3, 7, 17 aneuploidy; 9p21	Bladder carcinoma
HER-2/CEP17	17q11.2-q12	Breast cancer
TOP2A	17q21-22	Breast cancer
TERC/CEP3	3q26.3	Cervical cancer
EWSR1	22q12	Ewing sarcoma
EGFR	7p12	NSCLC
ALK	2p23	NSCLC
ROS1	6p22	NSCLC
TMPRSS2/ETV1	21q22.3/7p21.1	Prostate cancer
TMPRSS2/ETV4	21q22.3/17q21.3	Prostate cancer
ERG	21q22.2	Prostate cancer

## Abbreviations

FISH: Fluorescence in situ hybridization; CC: Conventional cytogenetics; aCGH: Array-based comparative genomic hybridization; SNP-arrays: Single nucleotide polymorphism arrays; CNAs: Copy number alterations.

## Competing interests

All authors declare that they have no conflicts of interests.

## Authors’ contributions

WM and KR wrote the article; LH, LZ, YH, XZ, HL and WM were involved in qmFISH research; AZ and TC reviewed the article. All authors read and approved the final manuscript.
